# Virtual Staining of Nonfixed Tissue Histology

**DOI:** 10.1016/j.modpat.2024.100444

**Published:** 2024-02-06

**Authors:** Nir Pillar, Yuzhu Li, Yijie Zhang, Aydogan Ozcan

**Affiliations:** a Electrical and Computer Engineering Department, University of California, Los Angeles, California;; b Bioengineering Department, University of California, Los Angeles, California;; c California NanoSystems Institute (CNSI), University of California, Los Angeles, California

**Keywords:** deep learning, frozen section, novel imaging modalities, surgical pathology, virtual staining

## Abstract

Surgical pathology workflow involves multiple labor-intensive steps, such as tissue removal, fixation, embedding, sectioning, staining, and microscopic examination. This process is time-consuming and costly and requires skilled technicians. In certain clinical scenarios, such as intraoperative consultations, there is a need for faster histologic evaluation to provide real-time surgical guidance. Currently, frozen section techniques involving hematoxylin and eosin (H&E) staining are used for intraoperative pathology consultations. However, these techniques have limitations, including a turnaround time of 20 to 30 minutes, staining artifacts, and potential tissue loss, negatively impacting accurate diagnosis. To address these challenges, researchers are exploring alternative optical imaging modalities for rapid microscopic tissue imaging. These modalities differ in optical characteristics, tissue preparation requirements, imaging equipment, and output image quality and format. Some of these imaging methods have been combined with computational algorithms to generate H&E-like images, which could greatly facilitate their adoption by pathologists. Here, we provide a comprehensive, organ-specific review of the latest advancements in emerging imaging modalities applied to nonfixed human tissue. We focused on studies that generated H&E-like images evaluated by pathologists. By presenting up-to-date research progress and clinical utility, this review serves as a valuable resource for scholars and clinicians, covering some of the major technical developments in this rapidly evolving field. It also offers insights into the potential benefits and drawbacks of alternative imaging modalities and their implications for improving patient care.

## Introduction

Traditional surgical pathology workflow requires tissue to be removed from the patient, then fixed, embedded, grossly measured, sectioned, stained, and examined by a pathologist using a light microscope. This conventional tissue preparation is a labor-intensive and costly process, requiring several manual steps performed by trained technicians. Preparing tissue for microscopic imaging usually takes hours to days to complete and involves multiple hazardous chemicals and reagents. Some clinical scenarios require histologic evaluation with a faster turnaround time. The most common one is intraoperative consultation, where histologic tissue analysis is expected within minutes to provide live surgical guidance. For example, in oncologic operations, the border of the removed tissue is often examined to confirm negative margins, meaning normal tissue surrounding the resected tumor. Additionally, morphologically suspicious tissues, detected as incidental findings during oncologic operations, are often sent to intraoperative pathologic evaluation to ensure they are not involved with tumors. Such intraoperative pathology consultations are currently performed using the frozen section (FS) technique, which typically includes cutting a small portion of fresh tissue sent for evaluation, embedding it in an optimal cutting temperature compound, followed by freezing the tissue. Next, the frozen tissue is cut into thin sections (5–8 mm) using a cryostat and stained with H&E for direct examination under a microscope. The pathologist then assesses the morphologic appearance of the tissue, as seen in an H&E slide, and then responds to the clinician’s query. The majority of FS block turnaround times can be performed within 20 to 30 minutes^1^ and require a team of skilled technicians and clinicians working together in a surgical pathology laboratory to produce and interpret slides. The rapid freezing and processing of tissue samples introduce technical artifacts that distort cell morphology, making it challenging to interpret microscopic images accurately and contributing to a 1.4% to 2.7% discordance rate between FS and the permanent sections.^2,3^ Overall, sampling problems and suboptimal quality sections are recognized in nearly a third of the FS cases.^4^ Furthermore, in cases where the tissue sample is limited, the inherent loss of tissue that occurs as part of FS preparation can potentially compromise the future ability to reach a definite diagnosis.

To address these challenges, a wide array of novel optical imaging modalities are being evaluated for their utilization in rapid microscopic tissue imaging, to achieve higher accuracy and shorter turnaround time than current intraoperative pathology practice. These modalities differ from each other by their optical characteristics (eg, spatial resolution, depth of field, and contrast mechanism), the tissue preparation required for imaging (fresh, rinsed, fast stained, or frozen), and the imaging equipment needed for their deployment (price, scanning time, size, staff training) and by their output (monochromatic cellular image, spectrogram, frequency graph, etc). As this field is evolving rapidly, it may prove challenging for practitioners to keep track of these developments and acquire sufficient experience in each specific modality. Pathologists are trained to identify tissue morphology and to provide histologic diagnosis based on the microscopic H&E appearance of cells and tissue. Hence, some of these new imaging methods have been recently combined with computational algorithms to transform the output of these emerging imaging modalities into H&E-like images, which is important to expedite the acceptance of these new techniques by eliminating alternative image output styles beyond the traditional training of pathologists. Although different reviews focusing on emerging imaging devices for intraoperative tissue analysis have been published,^5,6^ they primarily focused on imaging modalities, covering various nonstandard image formats outside of the traditional training of pathologists, and did not provide an overview of the specific clinical needs for virtual staining of nonfixed tissue from different organs.

**Figure 1.**
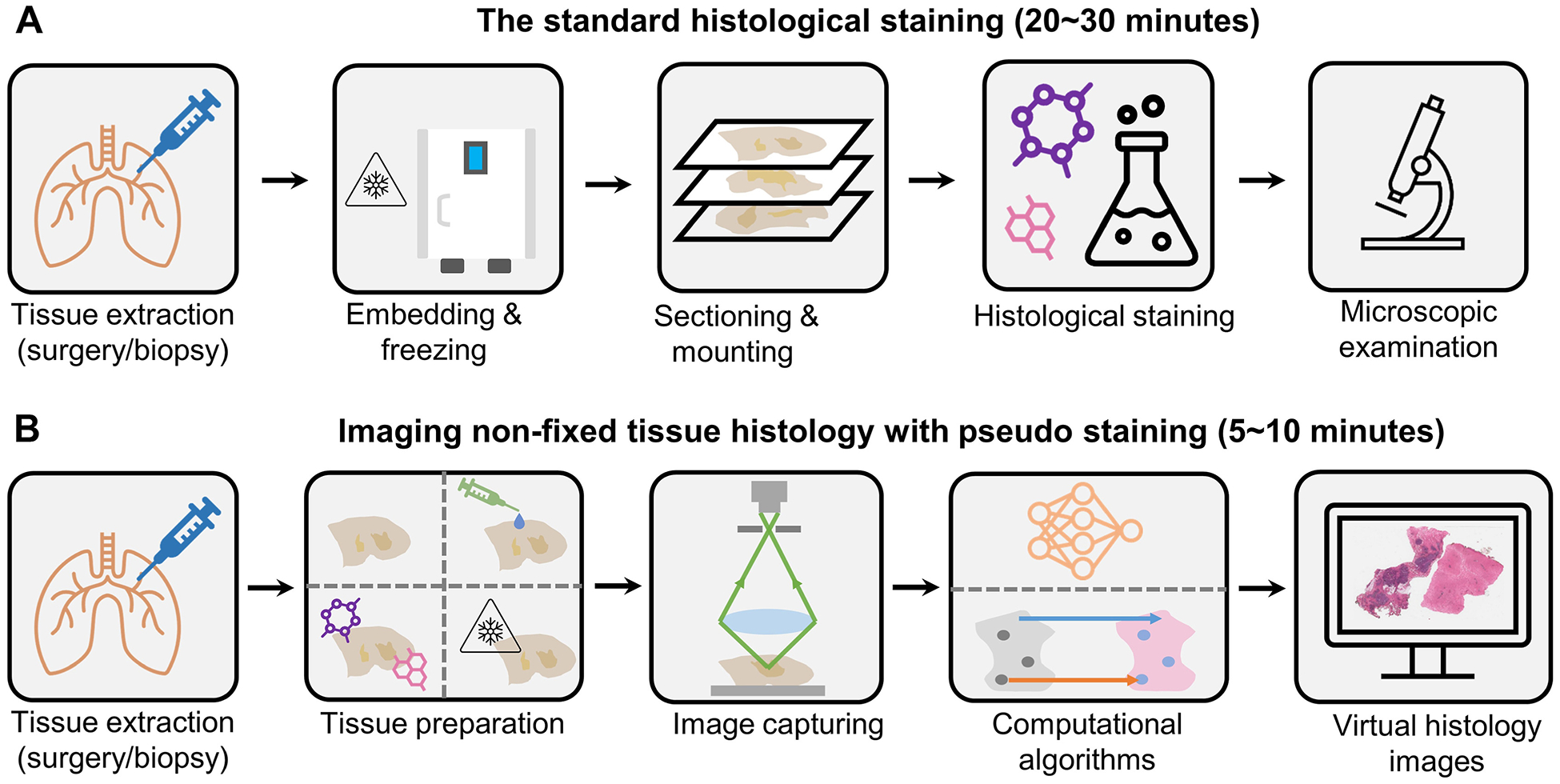
Schematic of the standard frozen section histologic staining and alternative imaging modalities for nonfixed tissue with hematoxylin and eosin–like virtual staining. (A) Standard histologic staining of nonfixed tissue requires several steps of tissue embedding, freezing, and labeling, with a mean turnaround time of nearly 30 minutes. (B) Alternative imaging modalities can significantly shorten the turnaround time for microscopic evaluation by utilizing various optical imaging modalities combined with digital staining algorithms.

In this review, we provide a comprehensive, anatomically oriented survey of emerging microscopy modalities for imaging nonfixed human tissue; our focus is on optical imaging techniques that specifically generate H&E-like images of nonfixed tissue samples that were evaluated by pathologists, bringing immediate clinical relevance to the output images of these techniques (Fig. 1). This comprehensive review will serve as an atlas of the recent technical developments in the field, offering an overview of the top-level concepts, up-to-date research progress, and the clinical utility of these advancements. We aimed to provide researchers, scholars, and clinicians with valuable insights and a clear understanding of the current state of the art in this rapidly evolving and transformative field.

## Methods

### Literature Search and Screening Process

A comprehensive, systematic literature search was performed in September 2023. PubMed, EMBASE, and Web of Science were searched using the following search strings in the “Title/abstract” field: “reflectance confocal microscopy” AND “H&E”, “nonlinear microscopy” AND “H&E”, “optical coherence tomography” and “H&E”, “fluorescence confocal microscopy” AND “H&E”, “light sheet microscopy” AND “H&E”, “photoacoustic microscopy” AND “H&E”, “multi-photon microscopy” AND “H&E”, “structured illumination microscopy” AND “H&E”, “pseudo H&E”, “virtual H&E”, “digital H&E”, and “digital staining”. The search was limited to articles in English, without publication time limits. All the titles and abstracts were checked by 2 different researchers (N.P. and Y.L.). Duplicates were removed, and all relevant articles were collected and studied. Relative bibliographies were hand-searched to identify further relevant literature. If there was a difference in opinion on the suitability of the works among the researchers, a consensus was reached by consulting a third senior reviewer (A.O.). Due to the large differences in methodologies and patient cohorts available in different studies, the literature search did not strictly follow the criteria for a systematic review. A stringent attempt was made to identify the highest quality of available evidence for each specific theme.

### Eligibility Criteria

After the screening process, the remaining articles were fully reviewed by 2 authors (N.P. and Y.Z.) to check their relevance and eventual accordance with the below-mentioned inclusion and exclusion criteria. Briefly, only studies concerning novel imaging applications of nonfixed human tissues compared with traditional histology were analyzed. The clinical results of works with both a preclinical and a clinical experimental part were also included. The following inclusion/exclusion criteria were followed:

### Inclusion Criteria:

1. Original peer-reviewed publications focusing on histologic imaging of nonfixed human tissues.
2. An image transformation step from the microscopic imaging input into an H&E-like image is provided.
3. A visual representation of the H&E-like stained images next to the histochemical H&E stain is available in this article.
4. A comparison between the H&E-like images and the histochemical images, conducted by certified pathologists, and statistical metrics of this comparison are provided.

### Exclusion Criteria:

1. Correspondences/Comments/Letters to the editor/Proceedings/Conference papers/Case reports/Reviews/Preprints

### Data Extraction

All the included studies were analyzed, and the following data were extracted and summarized: authors, publication year, number of patients/samples, specific imaging modality used, type of dye implemented (if used), information on the imaging instrument/commercially available or not, number of pathologists involved, diagnosis accuracy, and overall tissue imaging time. Considering the extreme heterogeneity of the studies available and the limited number of published works, we present the data as a comprehensive narrative review.

**Figure 2.**
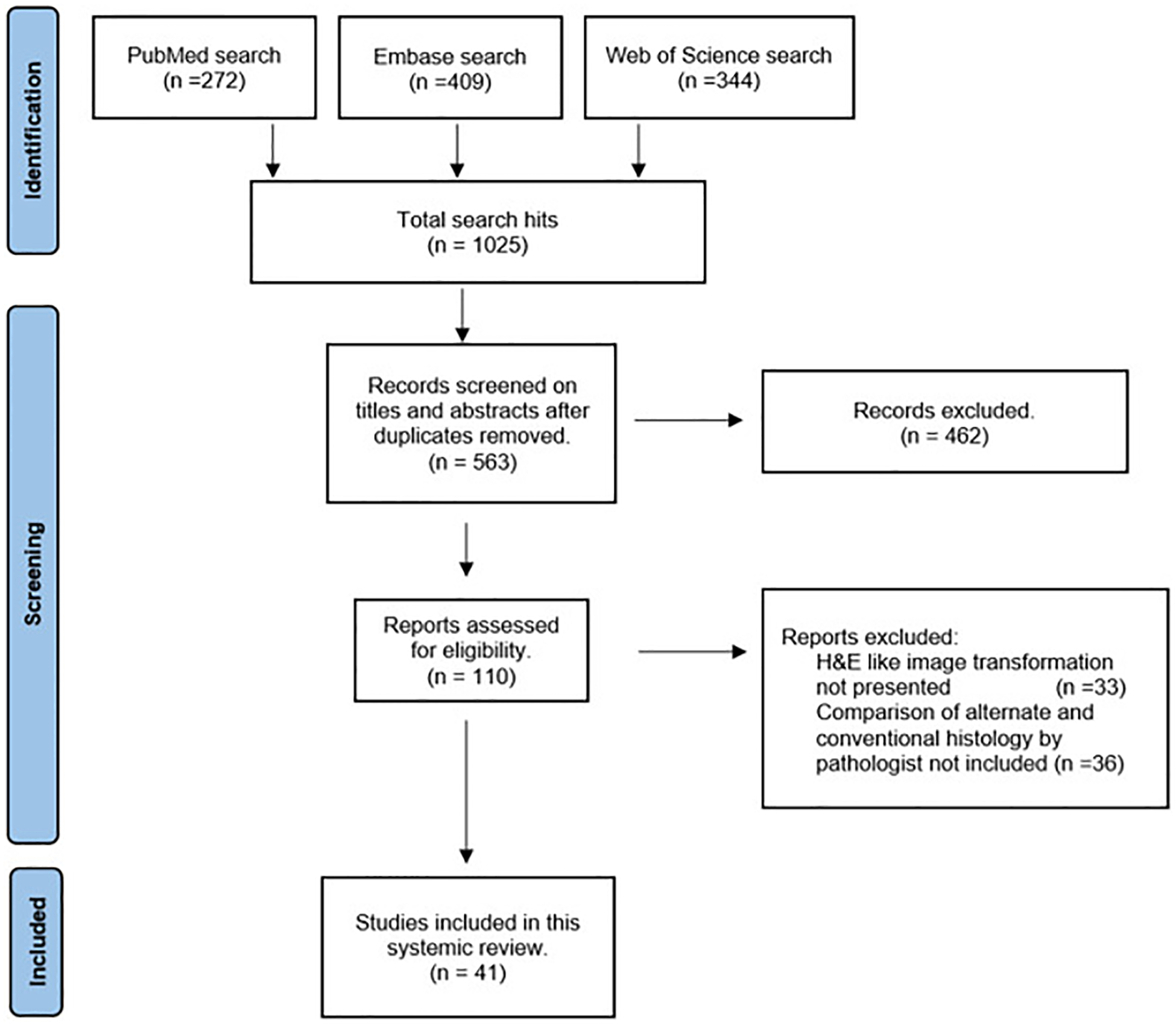
Study selection.

A total of 1025 hits were recorded by the first search among the 3 databases (PubMed 272, EMBASE 409, and Web of Science 344). Of these, 563 were screened reading titles and abstracts, removing duplicates. Next, 462 records were excluded (due to, eg, nonhuman tissues/fixed human tissues/no histology). Finally, 110 full-text articles were assessed for eligibility, finding 41 articles suitable for the review (Fig. 2).

## Results

### Skin

Skin cancer is among the most common cancers in men and women in the United States,^7^ and is classified as either non-melanoma skin cancer (ie, basal cell carcinoma [BCC] and squamous cell carcinoma [SCC]) or melanoma skin cancer. Approximately 5.4 million cases of nonmelanoma skin cancer are diagnosed annually in the United States.^8^ Although invasive melanoma is only a small percentage of all skin cancer cases, it is responsible for the vast majority of skin cancer deaths.^9^ Skin cancer screening through total body skin examination is arguably the safest and possibly one of the most cost-effective screening tests in medicine.^10^ BCC and SCC may be readily identified through visual inspection by a certified dermatologist. However, multiple benign lesions can mimic these cancers, resulting in unnecessary morbidity through invasive biopsies and treatments. Over 8.2 million skin biopsies are performed annually to diagnose over 2 million skin cancers in the Medicare population alone,^11^ emphasizing the amount of resources diverted into diagnosing various malignant skin lesions. These numbers, along with minimizing the removal of normal skin surrounding the lesion to optimize the cosmetic outcome, paved the way for major advancements in alternative diagnostic modalities. Initial reports of alternative imaging modalities in dermatology focused on the application of Mohs surgery, which requires obtaining fresh tissue and rapid diagnosis, and were later evaluated on skin biopsies and larger resections. Gareau et al^12^ utilized a fluorescence confocal microscope (FCM) combined with a reflectance confocal microscope (RCM) with a rapid staining protocol based on acridine orange to identify BCC resection margins and showed 81% accuracy, after discarding 40% of stained samples from their analysis due to poor quality. Mu et al^13^ demonstrated a higher diagnostic accuracy for the identification of nonmelanoma skin cancer (NMSC) and demonstrated how a 5-minute training period could improve the pathologic evaluation of the digitally stained FCM. However, over a quarter of the image samples were removed before the analysis due to uninterpretable image quality. Peters et al^14^ examined an FCM-based system with improved image acquisition that demonstrated similar BCC diagnostic accuracy and improved ease of use. 3% of the scanned images were discarded due to low image quality. Ortner et al^15^ investigated the utility of DRAQ5 staining compared to acridine orange staining and showed higher nuclear-specific staining, increased photostability, and better pseudo hematoxylin in digitally stained confocal mosaics. Ruini et al^16^ tested confocal microscopy on frozen tissue sections and reported its high concordance with histology. However, only medium concordance was noted for nonradical excisions. Li et al^17^ implemented a deep learning–based framework that uses a convolutional neural network to transform in vivo RCM images of unstained skin into virtually stained H&E-like images, introducing a “virtual biopsy” concept that allows for a histologic image to be synthesized based on intact skin imaging. Vladimirova et al^18^ tested confocal microscopy accuracy in a variable cohort of malignant and benign skin diseases and showed a high concordance with histology. Ching-Roa et al^19^ evaluated the utility of 2-photon fluorescence microscopy for imaging tissue samples and found high concordance with traditional histology while enabling deeper imaging depth than other fluorescent imaging techniques. Approximately 15% of the scanned images were removed from the analysis due to significant artifacts. Ogrzewalla et al^20^ tested FCM in a clinical setting, examining a wide array of epithelial tumors, melanocytic tumors, and inflammatory dermatoses. They reported an accuracy of 74%.

### Oral Cavity

The ability to surgically remove cancers with negative margins significantly impacts local tumor recurrence and overall survival for patients with head and neck cancers.^21^ Despite the recent advancements in both surgical techniques and medical imaging, positive margin rates in oral SCC remain the highest among solid malignancies and have not improved significantly over the past 2 decades.^22^ Due to the delicate nature of the head and neck region and the potential risks involved in expanding the surgical area or causing harm to vital structures, acquiring additional unaffected tissue during procedures is often undesirable. However, in cases where a pathologist, during an intraoperative consultation, determines that a surgical margin is “involved with tumor”, the surgeon can promptly proceed with additional tissue removal to achieve complete resection. This proactive approach enables the achievement of a satisfactory margin in a single surgical procedure. Consequently, FS analysis is significant in the context of head and neck surgeries. Shavlokhova et al^23^ demonstrated the potential of FCM in oral oncology and its ability to diagnose oral SCC with an accuracy of more than 95%, albeit with moderate specificity. In a follow-up work, Shavlokhova et al^24^ evaluated the utilization of FCM for detecting oral leukoplakia and showed FCM to be a clinically applicable alternative method for the digital diagnosis of mucosal dysplasia. They also provided diagnosticians with a series of confocal images with corresponding histopathological slides of oral mucosa specimens, including normal, dysplastic, and cancerous tissues.^25^ The agreement between the confocal imaging and ground truth histology was very good, with a mean accuracy of 94%. This accuracy was calculated based on a relatively narrow spectrum of histologic entities. Steybe et al^26^ imaged oral SCC samples using a stimulated Raman scattering (SRS) microscopy, with H&E pseudocoloring of SRS microscopic images, referred to as stimulated Raman histology (SRH). They compared their results with FS, demonstrating high diagnostic accuracy without requiring thin tissue sectioning or staining. However, the relatively low resolution of SRH compared with conventional H&E is currently preluding the accurate identification of precursor lesions.

### Digestive System

Despite its high prevalence, intraoperative FS is required only rarely in colorectal cancer resections and is usually ordered when lymph nodes appear suspicious for metastasis or unexpected liver subcapsular lesions not identified on preoperative imaging are noted by the surgeons.^27^ In low rectal cancer operations, FS is often performed for resection margin assessment in sphincter-preserving procedures^28^ and in certain cases, guides an intra-operative decision regarding the performance of a restorative proctectomy.^29^ Surgical margins are also often assessed during pancreatoduodenectomy, which was shown to dramatically increase the complete resection rate.^30^ Intraoperative FS investigations in the digestive tract are most commonly performed in hepatobiliary and pancreatic surgical procedures, where morphologically suspicious tissue fragments from the peritoneal cavity, liver, and lymph node specimens are sent for consultation.^31^ Another major use of FS evaluation of liver tissue is assessing the suitability of the liver for transplantation. Given the high prevalence of obesity, diabetes, and fatty liver disease globally, liver steatosis is a common risk factor for primary dysfunction after transplantation.^32^ To have an up-to-date histologic evaluation of the transplant, FS is often performed simultaneously with liver harvesting. Intraoperative FS is extremely important during the operative treatment of Hirschsprung disease, first to establish that ganglion cells are present and subsequently to ensure adequacy at the proximal surgical (anastomotic) margin.^33^ Sarri et al^34^ employed an SRH system to visualize freshly excised colon and pancreatic tissues, with benign and neoplastic findings. They reported high agreements between SRH and H&E images acquired on the same patients for healthy, precancerous, and cancerous colon and pancreas tissue sections. As previous SRH reports evaluated the agreement between SRH and H&E images on the same tissue but at different locations or on adjacent sections, Sarri et al^35^ performed one-to-one comparison on identical samples and showed good agreement between SRH and H&E images on identical tissues, however, with a long image acquisition time (eg, 25 minutes for 1 mm^2^ of tissue). Liu et al^36^ described an SRH-based system that demonstrated high diagnostic accuracy with a significantly shorter image acquisition time by using a deep learning–based algorithm. Their comparison with human pathologists demonstrated high accuracy in determining malignant and benign lesions and identifying tumor margins, including in the evaluation of resection margins of endoscopic submucosal dissection. Krishnamurthy et al^37^ used FCM to visualize core needle biopsies (CNB) of benign and malignant liver tissues and demonstrated high concordance with histology results. Follow-up, a prospective study by Krishnamurthy et al^38^ demonstrated the same methodology to perform real-time bedside evaluation of interventional radiology–guided liver CNB with high accuracy, compared with their histology counterparts.

### Breast

Intraoperative consultations are routinely performed during breast cancer operations, mostly to assess the status of axillary sentinel lymph node (SLN) involvement and to ensure patient suitability for breast-conserving surgery (validate free surgical margins in lumpectomies and confirm that no subareolar tumor involvement is identified in nipple-sparing mastectomies). If intraoperative SLN is positive, patients will proceed directly for immediate axillary lymph node dissection, thus sparing them from the burden of a second operation, which may be more complex, time-consuming, and carry greater risks.^39^ FS routinely performed during nipple-preserving mastectomies, where sub-areolar tissue is assessed for the presence of tumor cells, can help guide intraoperative reconstructive planning.^40^ Positive surgical margins are associated with a 2-fold increased risk for ipsilateral tumor recurrence.^41^ However, the assessment of intraoperative lumpectomy margins is not commonly performed, primarily due to the substantial increase in surgical duration caused by the intraoperative FS procedure, the limited diagnostic accuracy, and the freezing artifacts it introduces.^42^ Tao et al^43^ utilized modified nonlinear microscopy, consisting of second harmonic generation microscopy (SHG) and two-photon excitation microscopy (TPE) and demonstrated 94% overall accuracy in differentiating benign breast pathologies (fibroadenomas and benign breast parenchyma) from ductal carcinoma in situ (DCIS), lobular carcinoma in situ (LCIS), invasive ductal carcinoma (IDC), and invasive lobular carcinoma (ILC). Dobbs et al^44^ focused on inflammatory breast carcinoma and described a confocal microscopy-based framework to estimate the amount of invasive tumor cellularity directly from the CNB, demonstrating moderate interobserver agreement. Abeytunge et al^45^ applied a confocal strip microscope (a modified FCM of what was previously described by Dobbs et al^44^) that can rapidly scan large tissue sections and can be applied to surgical margin examination. They reported very good concordance with the corresponding histopathology. Over half of the scanned images were not included in the concordance analysis due to image acquisition pitfalls. Elfgen et al^46^ employed an FCM-based imaging system and demonstrated its capability to provide cellular visualization equivalent to H&E standards, thereby enabling swift and precise diagnosis of both malignant and benign breast tissue. Approximately 10% of the scanned images were removed before diagnostic analysis due to poor quality.

Krishnamurthy et al^37^ employed FCM to visualize 40 CNB of both benign and malignant breast tissue and showed an accuracy of 95% compared with histology. Lin et al^47^ applied a full-field optical coherence tomography–based microscope to intraoperatively evaluate the status of breast SLN biopsies. They demonstrated high concordance with the fixed tissue histologic assessment and matched accuracy compared with the FS results. Conversano et al^48^ evaluated the performance of pathologists in diagnosing cancerous and noncancerous breast tissue in ultrafast FCM and demonstrated diagnostic improvement after providing clinicians with an online training program.

### Central Nervous System

Most central nervous system (CNS) tumors do not have a distinct gross appearance and are sometimes inseparable from the adjacent normal brain parenchyma. Hence, neurosurgeons rely on intraoperative histopathologic evaluation in tumor identification and initial classification. However, the soft and friable nature of brain tumors makes them technically difficult to section in FS, and CNS tumor sections frequently show marked freezing artifacts.^49^ Smear (or squash) preparations are more rapid and offer both cytologic and architectural details, but they also exhaust the smeared piece of tissue and preclude permanent sections and any further studies on that exact tissue fragment. Orringer et al^50^ developed an SRH-based imaging system that demonstrated high accuracy and achieved a very good agreement with standard intraoperative histology techniques. This novel system enabled the identification of various CNS lesions without needing external stains while generating images comparable with traditional H&E staining. Hollon et al^51^ tested SRH on various pediatric-type CNS tumors and demonstrated high concordance with H&E diagnoses. Shin et al^52^ applied SRH to a diverse set of skull base tumors and demonstrated a high degree of agreement with H&E stains. Eichberg et al^53^ demonstrated a 30-minute reduction in intraoperative analysis of various CNS tumors by SRS-based microscope compared with FS with identical diagnostic accuracy. Hollon et al^54^ recently trained a convolutional neural network (CNN) classifier for CNS tumors based on their SRH morphology. They compared the diagnostic accuracy of pathologists comparing conventional intraoperative histology vs SRH images with CNN prediction, demonstrating a higher overall diagnostic accuracy of SRH+CNN vs H&E histology (94.6% vs 93.9%). Pekmezci et al^55^ examined glioma samples obtained from infiltrative tumor margins using SRH and demonstrated its putative usage in more complete glioma resections. Straehle et al^56^ quantified the neuropathological interpretability of SRH in a routine clinical setting without any specialized diagnostician training and prior expertise and reported a noninferior accuracy compared with FS. Reinecke et al^57^ performed a qualitative assessment of results from SRH+CNN vs H&E histology and reported a high concordance with the neuropathologist in distinguishing between tumor and benign tissues. Fitzgerald et al^58^ utilized SRH in sinonasal and skull base tumors, revealing very good concordance with histology in a broad array of malignancies. Hollon et al^59^ included an additional layer of molecular classification using artificial intelligence in SRH-based imaging. They demonstrated that SRH microscopy-based modality is capable of accurately predicting gliomas molecular alterations (IDH mutation, 1p19q co-deletion, and ATRX mutation) intraoperatively, in addition to creating H&E-like images, augmenting this diagnostic technique.

### Genitourinary System

Current evidence does not strongly support the benefit of routine FS on urologic cancer surgical margin specimens. Nevertheless, FS has been shown to reduce the incidence of positive surgical margins, particularly after radical prostatectomy and partial nephrectomy, at least in select patients.^60^ A meta-analysis has demonstrated that intraoperative FS had a very high diagnostic performance in detecting suspicious urethral and ureteral malignant involvement at the time of radical cystectomy.^61^ Apart from its utility in surgical oncology, intraoperative consultation is commonly performed during solid organ transplantation to validate the quality of the transplant and rule out any concerning issues that may arise during the transplant harvesting process. Kidney transplantation is the most frequently performed solid organ transplantation worldwide. It is not uncommon for pathologists involved in on-call rotations to be asked to classify lesions found during donor assessment and evaluate the kidneys’ suitability for transplantation using FS.^62^ Puliatti et al^63^ tested the level of agreement between FCM and histology of prostate biopsies, taken from radical prostatectomy specimens, and reported 91% accuracy. Cahill et al^64^ utilized SHG+TPE to detect carcinoma in fresh radical prostatectomies instead of paraffin-embedded H&E staining, without requiring tissue dissection into smaller sizes. The study demonstrated a high accuracy of 98.3%. In a subsequent study, Cahill et al^65^ evaluated SHG+TPE to prostate CNB of specimens from patients who underwent MRI-guided biopsy and also achieved high diagnostic accuracy. Titze et al^66^ utilized FCM as a screening tool for prostate tissue biobanking and demonstrated its accurate microscopic analysis of biobank samples before their cryopreservation. This approach ensures the inclusion of representative tumor and normal tissue samples for subsequent molecular analysis. Behr et al^67^ tested optical sectioning using structured illumination microscope (SIM) as guidance for localized ablation of prostate cancer. They showed the potential impact of intraoperative biopsy imaging on a novel “see-and-treat” workflow for applying ablation therapy for localized prostate cancer. They reported nearly perfect specificity, enabling the treating physician to expand tissue margins intraoperatively, ensuring more comprehensive coverage of the lesion and thereby improving oncologic outcomes. Almost 25% of the captured images were discarded before the analysis due to inconsistent quality. Mannas et al^68^ applied SRH to prostate biopsies and demonstrated accurate identification of prostate cancer in real time without the need for sectioning or tissue processing. Their approach may decrease the number of biopsies needed and potentially reduce the need for repeated procedures. Falahkheirkhah et al^69^ used SRS microscopy combined with deep learning-based virtual staining to obtain high-quality, thin-section diagnostic images from thick-cut, fresh frozen prostate tissues without stains. They demonstrated equal interobserver agreement between their virtual staining and histochemical ones. In addition, their virtual staining methodology allowed for the creation of a virtual fixed tissue appearance, which had relatively higher contrast and well-preserved morphology compared with nonfixed tissue morphology. Liu et al^70^ demonstrated the potential of optical sectioning based on SIM for the diagnosis of fresh, unfixed CNB to assist in histologic assessment of small renal masses rapidly. They showed an accuracy of 89.2% in differentiating benign from malignant lesions. Krishnamurthy et al^37^ used FCM to visualize benign and malignant renal tissues and reported 100% diagnostic concordance with traditional histology. Krishnamurthy et al^38^ conducted a subsequent prospective study that demonstrated FCM’s capability to produce images resembling H&E-stained sections to accurately diagnose renal lesions at the time of the biopsy. Villareal et al^71^ applied FCM for real-time assessment of non-neoplastic kidneys for clinical nephrology and transplant assessment. Their results demonstrate near-perfect agreement with histology on the conducted comparisons, including the number of glomeruli, glomerulosclerosis assessment, and inflammation quantification. However, the acridine orange stain applied prior to tissue scanning interferes with the examination of renal tissue under immunofluorescence. In the following work, Villareal et al^72^ evaluated FCM’s ability to recognize glomerular, tubulointerstitial, and vascular lesion patterns and reported that its diagnostic accuracy was noninferior to conventional histology. Nevertheless, their concordance was reported as “fair” for glomerular sclerosis, increased mesangial matrix and necrosis and “poor” for the evaluation of endocapillary and mesangial hyper-cellularity and tubular atrophy.

### Lung

FS was shown to provide useful histologic information in lung adenocarcinoma operations, which surgeons may consider when deciding the extent of surgical resection to perform.^73,74^ Determining tumor grade on FS offers high sensitivity and specificity; furthermore, it is associated with recurrence-free survival after sublobar resection.^75^ A meta-analysis reported the reliability of FS for identifying the invasion status of adenocarcinoma, with high diagnostic accuracy for differentiating noninvasive or minimally invasive tumors from invasive ones.^76^ Intraoperative diagnosis during lung operation can facilitate the identification of any unexpected findings, such as lymph node involvement or metastases, which can impact the surgical approach and postoperative management. As such, the use of intraoperative diagnosis in lung cancer surgery has become increasingly important in recent years. Krishnamurthy et al^37^ showed the feasibility of using an intraoperative FCM to distinguish malignant lung tissue from benign and showed an overall accuracy of 91.3%. A follow-up study by Krishnamurthy et al^38^ also demonstrated FCM’s capability to accurately diagnose lung lesions at the time of the biopsy.

## Discussion

Successful intraoperative histologic consultation, an integral part of surgical pathology, depends on several key factors, such as the collaboration between pathologists and surgeons, the skill and experience of the pathologist, sample preparation, and rapid turnaround time. Currently, most intraoperative consultations are performed using FS, which requires manual preparation of slides by skilled histotechnicians, takes 20 to 30 minutes to complete, has some diagnostic inconsistencies compared with permanent sections and can exhaust small tissues during the sectioning, precluding additional tests on these tissues. In recent years, numerous articles have been published with a shared objective of enhancing the standard procedures for intraoperative consultations. Despite demonstrating high morphologic and diagnostic agreement with the ground truth (fixed tissue histomorphology), and even surpassing FS accuracy in certain models, these applications have yet to be integrated into routine clinical practice. All the included articles based their conclusions on a relatively small number of samples, ranging from several dozen to a few hundred samples, originated in a single institute. Before these imaging technologies can be used in clinical pathology practice, they must be thoroughly proven on a large scale through appropriate clinical trials at multiple institutions. This effort should compare their quality, turnaround time, reproducibility, cost-effectiveness, and diagnostic noninferiority to standard histology. In this review, we highlighted 2 limiting factors for this comparison: (1) the presentation format used by the majority of these innovative imaging models deviates from the H&E stain commonly employed by pathologists to evaluate FS cases, and (2) the lack of evaluations conducted by human experts between the generated images and the ground truth.

**Figure 3.**
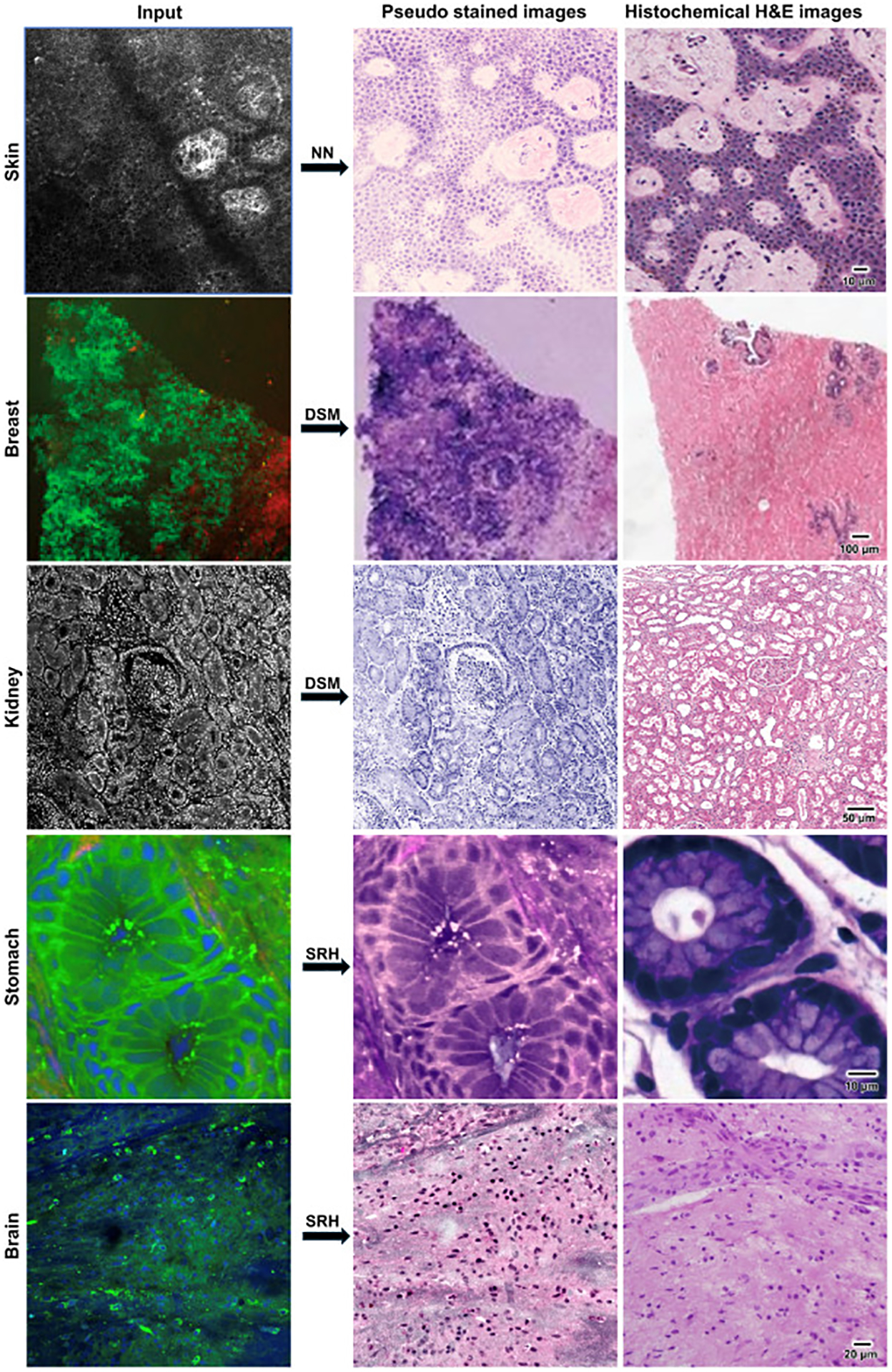
Representative tissue images with their pseudostained and conventional histology counterparts. Demonstration of pseudo staining in nonfixed tissue samples. Skin biopsy imaged with RCM and pseudostained with virtual staining.^17^ Breast specimen imaged using SHG+TPE and pseudostained with digital staining matching (DSM).^43^ Kidney samples imaged with combined FCM/RCM and pseudostained with DSM.^37^ Stomach imaged with SRS and pseudostained with SRH.^36^ Brain samples imaged with SRS and pseudostained with SRH.^50^

This review presented a comprehensive, anatomically organized summary of peer-reviewed articles that specifically focus on nonfixed tissue analysis. These articles present their imaging results in a format resembling H&E staining and incorporate a thorough comparison of these images against standard H&E images conducted by board-certified pathologists. The majority of included articles reported high degrees of accuracy between their pseudostains and the histologic counterparts, with a mean diagnostic accuracy exceeding 90%, as shown in Table 1.^12–20,23–26,34–38,43–48,50–58,63–72^ Additionally, the overall time required for image generation, which includes tissue positioning, staining, and rinsing, was significantly shorter, averaging less than 10 minutes, as detailed in Table 1. It is important to highlight that tissue size directly impacts imaging time. The majority of the included articles (35 of 41) utilized CNB or tissue fragments as input, and their speed advantages compared with FS need to be assessed in larger tissue sections as well. Furthermore, 2 articles^35,69^ attributed their longer-than-average imaging time to higher image quality. Representative comparisons of pseudostaining and histochemical H&E staining for nonfixed tissue samples are presented in Figure 3.^17,36,37,43,50^

To image these nonfixed tissue samples, several imaging modalities, as summarized in Table 2,^77–84^ were utilized. Notably, multimodal confocal microscopy modalities (RCM and FCM) were the most commonly used due to their ability to rapidly provide subcellular-resolution images without the need for physical sectioning of tissue samples. As evidence, more than half of the included articles in this review exploited either RCM, FCM, or a combination of both, especially in studies involving skin, breast, head and neck, and lung. Typically, RCM and FCM are deployed in tandem: the RCM primarily captures the features/signals from cellular cytoplasm and collagen, whereas the FCM is used for imaging nuclei. However, for the FCM mode, the autofluorescence signals from nonfixed tissue samples are usually insufficient to differentiate the nuclei from their surrounding cellular components. Therefore, a rapid staining process is typically required to highlight the nuclei features. The most investigated rapid stain for augmenting the nuclei contrast in FCM was acridine orange,^16,85–88^ which is a nucleic acid selective fluorescent dye. Its inherent affinity to nucleic acids allows it to produce distinct nuclear contrasts for nonfixed tissues by emitting fluorescent signals. These fluorescent signals can be detected and visualized using FCM, allowing for precise localization and 3-dimensional imaging of nucleic acids within the tissue samples.^86^ SRS-based modalities were the second most commonly used ones for imaging nonfixed tissue samples, predominant in CNS and gastrointestinal tract studies. They can create images based on the distinctive chemical bonds found in the cell cytoplasm and cell nuclei, providing a unique and intermediate label-free molecular contrast to identify tissue microscopic features. Moreover, other imaging modalities, such as TPE, SHG, and optical sectioning SIM, were also adopted, usually associated with acridine orange labeling to highlight nuclei features combined with a cytoplasmic stain such as sulforhodamine b^64,65^ or eosin Y^70^ to achieve higher image contrast. There are additional ex vivo imaging modalities being evaluated in various aspects of histopathology, such as microscopy with UV surface excitation (MUSE) and light sheet microscopy. However, no virtual staining or pathologists’ comparison of their output to intraoperative traditional histology has been published to date.

During our analysis of the various digital staining techniques employed by the articles included in our study to convert their imaging results into an H&E-like format (as detailed in Table 3^80,86,89,90^), we observed that most methods rely on fixed color mappings (eg, digital staining matching [DSM] and SRH). These mappings involved matching specific output signals from the imaging modalities to RGB color space, which encompass a spectrum ranging from nuclear hematoxylin to cytoplasmic eosin. DSM was applied in most of the articles we reviewed, being the standard method in confocal microscopy-based (RCM and FCM) articles. DSM is based on the notion that different tissue components have different optical properties. Hence, this algorithm combines the captured images from FCM (corresponding to cell nuclei) and RCM (corresponding to cell cytoplasm and collagen), normalizes them, and converts each pixel into an H&E-like image using predefined RGB color vectors.^86^ Although this creates a similar image to histochemical H&E, many cellular and cytoplasmic features present larger variations than ground truth histology. Another typical technique also employing fixed color mapping is SRH, which is used to generate virtual images reminiscent of H&E staining for SRS microscopy. SRH is achieved by applying a simple linear color mapping between 2 channels captured by the SRS microscope. These 2 channels represent 2 Raman shifts: one at 2845/cm is associated with plentiful CH2 bonds found in lipids (corresponding to cell cytoplasm), whereas another at 2930/cm indicates CH3 bonds in proteins and DNA (corresponding to cell nuclei).^34^ This configuration furnishes abundant information for crafting H&E-like images.

More than 70% of the included articles in this review employed commercially available imaging devices that use a stain-matching algorithm to translate the acquired image data into H&E-like colors. The most commonly used ones in the included articles were Vivascope^91^ and Histolog^92^ scanners, which use DSM, and Nio imaging, which uses SRH. Vivascope 1500 and 3000 are FDA-cleared for noninvasive, in vivo skin imaging. These devices consistently demonstrated both high diagnostic accuracy and significantly reduced imaging time compared with traditional intraoperative FS. This suggests that these commercially available devices are reliable, user-friendly, and readily accessible in research centers where these studies were conducted. However, it is important to acknowledge that incorporating a new imaging device into clinical practice presents certain challenges. Creating a new imaging device requires close collaboration among clinicians, engineers, optical engineers, and computer scientists, as well as substantial financial resources (the cost of some of these novel imaging modalities is well over $100K).

An interesting observation emerged from this review, revealing a discrepancy between the frequency of FS use based on anatomical location and the use of alternative imaging techniques with pseudostaining to evaluate these specific indications. For example, FS performed during gynecologic operations (mostly operations for ovarian/endometrial carcinoma) constitute 10% to 20% of all performed FS.^93–95^ However, despite our efforts, we were unable to locate any research articles that met our inclusion criteria in this particular context. Although the exact reason for this scarcity of literature remains unclear, we speculate that it might be associated with the timing of FS during gynecologic operations. In such procedures, the FS is typically conducted at the beginning of the operation, after which the surgeon proceeds with the planned surgical intervention for more than 30 minutes. Consequently, there is no operational delay while the FS is being processed and examined in the pathology laboratory. This stands in contrast to other organ systems, such as the brain, breast, or gastrointestinal tract, where the operational procedure often pauses temporarily while the nature of the sampled lesion is being assessed by FS. It is plausible that the uninterrupted flow of the surgical procedure in gynecologic operations allows for immediate continuation, partially reducing the need for additional imaging techniques utilizing pseudostaining.

Three-dimensional histology, a field that enables tissue histology visualization at multiple depths, has the potential to improve diagnostic yield in many areas of pathology, allowing a more accurate representation of tissue heterogeneity. In this review, we cover 2 articles that described their ability to highlight tissue variations at different depths. Cahill et al^65^ used nonlinear microscopy to highlight perineural invasion and nerve free of carcinoma at different tissue depths, and Falahkheirkhah et al^69^ applied SRH to demonstrate tumor-free areas with the appearance of tumors on deeper sections. However, it is important to note that, in these described examples, the provided variations did not change the diagnosis status.

Li et al^17^ and Falahkheirkhah et al^69^ introduced a distinct staining methodology in their studies employing a deep learning–based approach instead of a fixed color mapping, setting it apart from other articles. Their presented virtual staining methods used CNN to learn the mapping between RCM images (Li et al^17^), SRS images (Falahkheirkhah et al^69^), and H&E-stained tissue images. Falahkheirkhah et al utilized their virtual staining methodology on SRS images to create a virtual fixed tissue appearance, with relatively higher contrast and well-preserved morphology compared with nonfixed tissue morphology. Li et al^17^ trained a CNN to generate virtual H&E-stained tissue images from RCM images of in vivo skin lesions in a noninvasive manner, creating “virtual biopsies”.

Intraoperative consultations performed on in situ tissues are currently being investigated across various surgical oncology disciplines, including head and neck,^96^ gastrointestinal,^97^ CNS,^98^ lung,^99^ breast,^100^ and ovaries.^87^ However, these innovative modalities have not yet incorporated a histopathological presentation resembling H&E staining for their results. Additionally, they do not encompass a pathologist’s comparison between the in vivo imaging results and the ground truth histology. One significant factor contributing to this limitation is the inability to directly compare identical regions between in vivo imaging and ex vivo tissue analysis. This challenge arises due to the complex and invasive nature of the biopsy and H&E histochemical staining procedures, which involve a series of intricate and destructive operations. It is conceivable that in the future, a virtual staining methodology, akin to the one outlined for skin, may be developed and utilized for various other organs.

## Summary

Intraoperative pathology consultations play a crucial role in ensuring precise tissue diagnosis. However, FS, the common clinically approved method for performing these consultations, presents significant challenges. FS heavily depends on dedicated trained personnel for execution, demanding an extensive duration of 20 to 30 minutes to complete, and can lead to a notable depletion of tissue, which is especially relevant in small tissue biopsies. In this study, we presented a comprehensive anatomically based evaluation of alternative imaging modalities to FS. These modalities were assessed based on their ability to generate pseudo-H&E–stained outputs, which were then compared with ground truth histology by experienced pathologists. Nearly all selected articles presented high diagnostic accuracy in their approaches, paving the way for additional wide evaluation before their clinical acceptance. We predict that the number of alternative imaging modalities, which include H&E-like stained outputs that are computationally generated, will significantly increase future shortly, potentially becoming mainstream for intraoperative diagnosis.

## Figures and Tables

**Table 1:** Selected articles on nonfixed tissue imaging

Anatomical location	Publication	Samples tested	Goal of study	Optical modality	Stain used	H&E-like methodology	Commercial?	No, of pathologists	Overall accuracy compared to histology	Time to image analysis
SK	Garaeu et al, 2012^12^	21 BCCs	Identify BCC on resection margin	Combined RCM and FCM	Acridine orange	DSM	Vivascope 2000	1	81% accuracy	2–10 minutes
SK	Mu et al, 2016^13^	64 samples (NMSC and normal skin)	Differentiate NMSC from benign skin	Combined RCM and FCM	Acridine Orange and Eosin	DSM	Vivascope 2500	3	Sens-98% Spec-96%	5–10 minutes
SK	Peters et al, 2019,^14^	148 BCCs	Detection of BCCs	Combined RCM and FCM	Proflavine solution	DSM	Histolog Scanner	2	Sens-73% Spec-96%	2–15 minutes
SK	Ortner et al, 2021^15^	8 BCCs	Compare DRAQ5 to acridine orange staining	Combined RCM and FCM	DRAQ5	DSM	VivaScope 2500	3	82% agreement	5–10 minutes
SK	Ruini et al, 2021^16^	46 BCCs	Test imaging on frozen skin with BCC	Combined RCM and FCM	Acridine orange	DSM	VivaScope 2500	2	Sens- 88% Spec-100%	5–10 minutes
SK	Li et al (in vivo), 2021^17^	8 BCCs	To create virtual histologic images based on in vivo RCM	RCM	None	Label-free virtual staining	VivaScope 1500	2	Sens-80% Precision-70%	2–5 minutes
SK	Vladimirova et al, 2022^18^	120 samples (BCC, SCC, multiple myeloma and benign skin diseases)	Test confocal imaging on various skin malignancies	Combined RCM and FCM	Acridine Orange	DSM	VivaScope 2500M-G4	2	89% accuracy	5–10 minutes
SK	Ching-Roa et al, 2022^19^	15 NMSCs	Can a 2-photon fluorescence microscope enable rapid diagnosis of NMSC	Two-photon fluorescence microscopy	Acridine orange and Eosin	DSM	No	2	Sens-93% Spec-100%	2–3 minutes
SK	Ogrzewalla et al, 2022^20^	66 samples from 55 patients (epithelial tumors, melanocytic tumors, and benign lesions)	Compare FCM to histology in a clinical routine setting	Combined RCM and FCM	Acridine orange	DSM	Vivascope 2500	2	74% Accuracy	5–10 minutes
HN	Shavlokhova et al, 2020^23^	70 oral lesions from 70 patients	Validate the diagnostic accuracy of FCM against histopathology	Combined RCM and FCM	Acridine orange	DSM	Vivascope 2500	1	96.7% accuracy	2–5 minutes
HN	Shavlokhova et al, 2021^24^	27 oral lesions from 22 patients	Analyze the sensitivity and specificity of FCM for detecting oral leukoplakia, compared with histopathology	Combined RCM and FCM	Acridine orange	DSM	Vivascope 2500	1	94.3% accuracy	2–5 minutes
HN	Shavlokhova et al, 2022^25^	50 oral mucosa samples including oral SCC, dysplasia, and healthy oral tissue	Create an image atlas of benign and malignant oral tissues and compare them to the corresponding histopathology	Combined RCM and FCM	Acridine orange	DSM	Vivascope 2500	2	94% accuracy	2–5 minutes
HN	Steybe et al, 2023^26^	80 tissue samples from 8 patients	Evaluate the potential of SRH for the analysis of oral SCC compared to FS	SRS	None	SRH	Nio imaging	2	Sens-100%, Spec- 90.1%	5–18 minutes
GI	Sarri et al, 2019^34^	Colon, pancreas, and gastric samples. Number not included	Enable SRH cancer detection in unprocessed tissues and its histology comparison	SRS	None	SRH	No	1	92% Accuracy	25 minutes
GI	Sarri et al, 2019^35^	Colon, pancreas, and gastric samples. Number not included	Evaluate SRH/H&E correlation on the same tissue section	SRS	None	SRH	No	1	93.6% accuracy	25 min
GI	Liu et al, 2022^36^	279 samples 103 with gastric cancer and 176 with benign gastric tissue	Test SRH in intraoperative gastroscopy and its concordance with H&E	SRS	None	SRH	No	4	96% accuracy	2 min
GI	Krishnamurthy et al, 2019^37^	16 samples (benign liver, adenocarcinoma, and hepatocellular carcinoma)	Evaluate the quality of FCM for rapid and accurate evaluation of CNB	Combined RCM and FCM	Acridine orange	DSM	Vivascope 2500 RSG4	1	100% accuracy	5–10 min
GI	Krishnamurthy et al, 2020^38^	13 liver samples	Compare the accuracy of FCM CNB fresh biopsies to histology	Combined RCM and FCM	Acridine orange	DSM	Vivascope 2500 RSG4	2	96.2%	5–10 min
BR	Tao et al, 2014^43^	138 Samples with normal breast tissue, fibroadenomas, LCIS, DCIS, IDC, and ILC	Assess the suitability of SHG+ TPE for rapid intraoperative breast specimen	SHG and TPE	Acridine orange	DSM	No	3	Accuracy- 94.1%	5–10 min
BR	Dobbs et al, 2015^44^	23 CNB, clinically suspected inflammatory breast cancer	Evaluate sampling adequacy in inflammatory breast carcinoma	FCM	Proflavine	DSM	Vivascope 2500	1	Kappa agreement 0.48	2 min
BR	Abeytunge et al, 2017^45^	18 Samples (IDC, DCIS, and benign lesions)	Evaluate large specimen imaging quality compared to histology	FCM	Acridine orange	DSM	No	1	88.2% accuracy	<10 min
BR	Elfgen et al, 2019^46^	24 Samples that presented as suspicious for cancer	Evaluate the correspondence of breast cancer diagnosis between the assessment of confocal HS images and gold standard histologic images	Combined RCM and FCM	Acridine Orange	DSM	Histolog scanner	2	95% accuracy	2–5 min
BR	Krishnamurthy et al, 2019^37^	40 Breast CNB (23 benign, 17 malignant)	Evaluate the quality of FCM for rapid and accurate evaluation of small tissue fragments	Combined RCM and FCM	Acridine orange	DSM	Vivascope 2500 RSG4	1	95% accuracy	5–10 min
BR	Lin et al, 2022^47^	29 Sentinel lymph node biopsies (average of 2.9 lymph nodes per biopsy)	Evaluate the utilization of FF-OCT based system for breast cancer lymph node metastasis assessment	FF-OCT	Hoechst	DSM	AcuOnPath	1	98.8 % accuracy	30–40 min
BR	Conversano et al, 2023^48^	181 Breast specimens from 181 patients	Prospective study of evaluation of physicians’ performance to blindly distinguish noncancerous and cancerous tissue	Combined RCM and FCM	Acridine orange	DSM	Histolog Scanner	3	99.6% accuracy	4–5 min
CNS	Orringer et al, 2017^50^	101 Samples (low- and high-grade glial tumors, meningiomas, metastases)	Differentiate among diagnostic classes of brain tumors	SRS	None	SRH	Nio imaging	3	94.3% accuracy	2–5 min
CNS	Hollon et al, 2018^51^	25 Samples (20 pediatric-type brain tumors and 5 normal specimens from epilepsy operations)	Obtaining histopathologic diagnosis in tumors common in the pediatric population	SRS	None	SRH	Nio imaging	3	95% accuracy	2–5 min
CNS	Shin et al, 2019^52^	16 Samples (meningiomas, schwannomas, chordoma, papillary craniopharyngioma)	Differentiate skull base tumors from benign tissue	SRS	None	SRH	No	3	87% accuracy	5–10 min
CNS	Eichberg et al, 2019^53^	82 Samples (gliomas, meningiomas, metastasis, pituitary adenoma, necrosis, normal brain)	Test for time to diagnosis of SRS vs FS in intraoperative neuropathology consultation	SRS	None	SRH	Nio imaging	1	91.5% accuracy	2–5 min
CNS	Hollon et al, 2020^54^	278 Samples	Evaluate CNN-based diagnosis of SRH images to pathologist-based interpretation of conventional histology	SRS	None	SRH	Nio imaging	2	93.9% accuracy	2–5 min
CNS	Pekmezci et al, 2021^55^	179 Surgical margin simples from 31 gliomas	Test SRH’s ability to facilitate more complete surgical resection	SRS	None	SRH	Nio imaging	3	Kappa agreement 0.72 between pathologists	2–5 min
CNS	Straehle et al, 2022^56^	73 Samples (gliomas, meningiomas, metastasis, reactive gliosis, pituitary, and adenomas)	Quantify SRH image’s interpretability and diagnostic accuracy	SRS	None	SRH	Nio imaging	1	87.7% accuracy	2–5 min
CNS	Reinecke et al, 2021^57^	94 Samples (gliomas, meningiomas, metastasis, lymphomas, and other, rarer primary brain tumors)	Evaluate SRS-CNN accuracy in the detection of tumors and differentiation from nontumorous brain	SRS	None	SRH	Nio imaging	1	Kappa agreement 0.9 between CNN and neuropathologist	2–5 min
CNS	Fitzgerald et al, 2022^58^	12 Samples (SCC, metastasis, rhabdomyosarcoma, papilloma, and adenoid cystic carcinoma)	Assess the accuracy of SRH in a range of sinonasal and skull base tumors	SRS	None	SRH	Nio imaging	1	93.8% accuracy	2–5 min
GU	Puliatti et al, 2019^63^	89 Core biopsies from 13 radical prostatectomies	Evaluate the diagnostic agreement compared to histology	Combined RCM and FCM	Acridine orange	DSM	Vivascope	3	91% accuracy	5–10 min
GU	Cahill et al, 2020^64^	122 Prostate samples from 40 radical prostatectomies,	Evaluate the usage of SHG+ TPE for intraoperative evaluation in radical prostatectomy	SHG and TPE	Acridine orange and sulforhodamine b	Virtual Transillumination Microscopy	No	3	98.3% accuracy	5–10 min
GU	Cahill et al, 2022^65^	170 Biopsies were collected from 63 patients	Test SHG+ TPE accuracy and procedure time in prostate CNB analysis	SHG and TPE	Acridine orange and sulforhodamine b	Virtual Transillumination Microscopy	No	3	95.5% accuracy	5–10 min
GU	Titze et al, 2022^66^	127 Punch biopsies from 40 prostatectomies	Evaluate FCM’s ability to analyze biobank samples before cryopreservation	FCM	Acridine orange	DSM	Vivascope 2500	2	96.8% accuracy	5–11 min
GU	Behr et al, 2023^67^	46 Prostate biopsies	Evaluating optical sectioning SIM as a method for on-site diagnostic biopsy imaging tool	optical sectioning SIM	Eosin Y and DRAQ5	DSM	No	2	89.5% accuracy	2–5 min
GU	Mannas et al, 2023^68^	32 Samples from 23 prostatectomies	Assess SRH utilization for rapid classification of prostate biopsies	SRS	None	SRH	Nio imaging	3	95.7% accuracy	2–5 min
GU	Falahkheirkhah et al, 2023^69^	75 Prostate samples	Combine SRS and virtual staining to facilitate faster pathologic evaluation	SRS	None	Label-free virtual staining	No	5	Kappa agreement 0.496 for tumor grading	10–14 min
GU	Liu et al, 2016^70^	65 Renal CNB for 19 patients	Differentiate neoplastic from benign kidney	optical sectioning SIM	Eosin Y and DRAQ5	DSM	No	1	89.2% accuracy	5–10 min
GU	Krishnamurthy et al, 2019^37^	39 Kidney fragments (23 normal, 2 benign tumors, and 15 malignant tumors)	Evaluate the quality of FCM for rapid and accurate evaluation of small tissue fragments	Combined RCM and FCM	Acridine orange	DSM	Vivascope 2500 RSG4	1	100% accuracy	5–10 min
GU	Krishnamurthy et al, 2020^38^	15 Renal biopsies	Compare the accuracy of FCM CNB fresh biopsies to histology	Combined RCM and FCM	Acridine orange	DSM	Vivascope 2500 RSG4	2	96.2% accuracy	5–10 min
GU	Villareal et al, 2021^71^	24 Renal samples from autopsies	Explore the feasibility of a fusion mode confocal microscope in the analysis of non-neoplastic kidney biopsies	Combined RCM and FCM	Acridine orange	DSM	Vivascope 2500	2	Kappa agreement 0.6–1 between pathologists	3–7 min
GU	Villareal et al, 2023^72^	48 Renal biopsies	Explore the feasibility of a fusion mode confocal microscope in the recognition of glomerular, tubulointerstitial, and vascular lesional patterns	Combined RCM and FCM	Acridine orange	DSM	Vivascope 2500	2	Kappa agreement 0.7–0.88	3–7 min
LU	Krishnamurthy et al, 2019^37^	23 Lung fragments (14 normal, 9 malignant)	Evaluate the quality of FCM for rapid and accurate evaluation of small lung tissue fragments	Combined RCM and FCM	Acridine orange	DSM	Vivascope 2500 RSG4	1	91.3% accuracy	5–10 min
LU	Krishnamurthy et al, 2020^38^	15 Lung biopsies	Compare the accuracy of FCM CNB fresh lung biopsies to histology	Combined RCM and FCM	Acridine orange	DSM	Vivascope 2500 RSG4	2	96.2%	5–10 min

BCC, basal cell carcinoma; BR, breast; CNB, core needle biopsy; CNS, central nervous system; DCIS, ductal carcinoma in situ; DSM, digital staining matching; FCM, fluorescence confocal microscope; FF-OCT, full-field optical coherence tomography; GI, gastrointestinal and liver; GU, prostate and kidney; HN, head and neck; HS, histochemical; IDC, invasive ductal carcinoma; ILC, invasive lobular carcinoma; LCIS, lobular carcinoma in situ; LU, lung; NMSC, non-melanoma skin cancer; RCM, reflectance confocal microscope; SCC, squamous cell carcinoma; SHG, second harmonic generation microscopy; SIM, structured illumination microscopy; SK, skin; SRH, stimulated Raman histology; SRS, stimulated Raman scattering microscopy; TPE, two-photon excitation microscopy.

**Table 2:** Comparison of imaging modalities

Modality	Mechanism	Tissue penetration	Spatial resolution
FCM/RCM	A laser beam is focused onto a single point within the sample, and the resulting fluorescence (FCM) or reflected light (RCM) is collected through a pinhole aperture to eliminate out-of-focus light. By sequentially scanning multiple points within the sample, a 2- or 3-dimensional image can be reconstructed.	10–50 mm^77^	Lateral: 200–1000 nm Axial: 2–5 μm^77^
SHG	A type of nonlinear microscopy that exploits the second harmonic generation effect to generate images. SHG occurs when 2 photons with the same frequency are combined in the optically nonlinear medium and then create an SHG photon with a frequency exactly twice the excitation frequency. Due to the anisotropic structure of collagen fibers, which is suitable for generating SHG signals, SHG microscopy is widely employed for imaging collagen and cytoplasm within tissue samples.	100–300 μm^78^	Lateral: 200–500 nm Axial: 1–1.5 μm^78^
TPE	A specific technique within nonlinear microscopy that leverages 2 photons with longer wavelength to excite fluorescent molecules. Because the photon flux is only sufficient around the focal point to trigger 2-photon absorption events, the resulting emission is confined to a small ellipsoidal volume surrounding this focal point. This effectively suppresses the emission of light from out-of-focus regions.	100–400 μm^79^	Lateral: 200–500 nm Axial: 1–1.5 μm^79^
SRS	A type of nonlinear microscopy that uses the stimulated Raman scattering (SRS) effect to generate images. In SRS, 2 laser beams of different frequencies (pump beam and stokes beam) are directed onto a sample. When the discrepancy between these 2 frequencies aligns with a specific molecular vibrational frequency, the intensity of the Stokes beam experiences an increase while the intensity of the pump beam experiences a loss. This intensity changes recorded by the detector are proportional to the concentration of the molecules within the sample, allowing SRS microscopy to be used for visualizing the spatial distribution of molecules in a sample.	10–100 μm^80^	Lateral: 130–500 nm Axial: 2–5 μm^81^
OCT	Light from a low-coherence light source is split into 2 paths: a sample arm containing the specimen and a reference arm that is typically a mirror. The combination of reflected light from the sample arm and reference light from the mirror give rise to an interference pattern. The measured interference pattern is used to create a depth profile of the sample.	3–5 mm^82^	Lateral: 5–20 μm Axial: 3–15 μm^82^
Optical sectioning SIM	Tissue specimens are illuminated with a periodic pattern of light intensity, typically a grid or sinusoidal pattern. By acquiring a series of images with structured illumination pattern with different phases, an optically sectioned, in-focus image of the sample can be reconstructed.	200 μm^83^	Lateral: 200–1000 nm Axial: 2–5 μm^83^
Bright-field microscope	This is a conventional and the simplest optical microscopic modality that pathologists use to observe stained samples. Samples are typically illuminated with white light, and the contrast is achieved through the attenuation of transmitted light in dense areas of the samples.	100 μm^84^	Lateral: 200–1000 nm Axial: ~3–5 μm^84^

FCM, fluorescence confocal microscope; OCT, optical coherence tomography; RCM, reflectance confocal microscope; SHG, second harmonic generation microscopy; SIM, structured illumination microscopy; SRS, stimulated Raman scattering microscopy; TPE, 2-photon excitation microscopy.

**Table 3:** Description of alternative staining modalities

Staining modality	Description
DSM^86^	An algorithm that digitally stains the grayscale fluorescence-only (nuclei) and reflectance-only (cytoplasmic collagen) images using predefined RGB color codes and then combines them into an H&E-like image. This algorithm is typically utilized for combined FCM and RCM imaging modality. The predefined RGB color codes for hematoxylin (resulting in a purple color in bright-field images) and eosin (resulting in a pink color in bright-field images) were usually experimentally determined by thoroughly sampling the purple/pink colors from histochemically stained H&E images. These RGB color codes of H&E are then weighted by the fluorescence-only (nuclei) channel and reflectance-only (collagen) channel before being combined, yielding a H&E-like image in which nuclei appear purple, whereas collagen and cytoplasm appear pink. The algorithm is based on the principle that different tissue components exhibit distinct optical properties. For instance, nuclei would emit fluorescence light when excited with ultraviolet light, whereas collagen and cytoplasm would backscatter or reflect light in the visible spectrum.
Label-free virtual staining^89^	A deep learning–based technique that allows the generation of a stained image of a biological sample without the use of any exogenous labels or stains. Instead of relying on specific labeling molecules or dyes, label-free virtual staining digitally utilizes intrinsic contrast mechanisms within the sample itself to produce a color image that mimics the appearance of a stained sample. It involves a one-time process step consisting of acquiring and processing a large volume of data, as well as carefully designing and training the neural networks. Once a satisfactory label-free virtual staining model is obtained and validated, its blind inference is rapid and repeatable.
SRH^80^	Images acquired from the SRS microscope undergo a simple linear color mapping of each channel. Following channel subtraction and flattening, linear color remapping is applied to 2 SRS channels, the 2845/cm channel and the 2930/cm channel. The 2845/cm image (corresponding to cell cytoplasm), a grayscale image, is linearly mapped to an eosin-like color. A similar linear mapping is applied to the 2930/cm image (corresponding to nuclei) with a hematoxylin-like color assigned to a strong signal. Finally, these 2 channels are linearly combined to produce the final virtually colored H&E image.
Virtual transillumination microscopy^90^	The model uses a physically realistic rendering approach based on modeling transillumination absorption using the Beer–Lambert law. The model works by first calculating the absorption and scattering coefficients for each pixel in the image. These coefficients are then used to calculate the amount of light that reaches the camera from each pixel in the tissue. The light intensities are then analogously mapped to H&E-like colors.

DSM, digital staining matching; SRH, stimulated Raman histology.
